# Capability of common SNPs to tag rare variants

**DOI:** 10.1186/1753-6561-5-S9-S88

**Published:** 2011-11-29

**Authors:** Xiangqing Sun, Junghyun Namkung, Xiaofeng Zhu, Robert C Elston

**Affiliations:** 1 Department of Epidemiology and Biostatistics, Case Western Reserve University, Cleveland, OH 44106, USA

## Abstract

Genome-wide association studies are based on the linkage disequilibrium pattern between common tagging single-nucleotide polymorphisms (SNPs) (i.e., SNPs having only common alleles) and true causal variants, and association studies with rare SNP alleles aim to detect rare causal variants. To better understand and explain the findings from both types of studies and to provide clues to improve the power of an association study with only common SNPs genotyped, we study the correlation between common SNPs and the presence of rare alleles within a region in the genome and look at the capability of common SNPs in strong linkage disequilibrium with each other to capture single rare alleles. Our results indicate that common SNPs can, to some extent, tag the presence of rare alleles and that including SNPs in strong linkage disequilibrium with each other among the tagging SNPs helps to detect rare alleles.

## Background

In recent years, genome-wide association studies have identified hundreds of genetic variants that may be associated with many common diseases [1-3]. It is believed that the associated single-nucleotide polymorphisms (SNPs) detected from current association studies may represent linkage disequilibrium (LD) between a common tagging SNP and true causal variants. Under the common disease/rare variants hypothesis, which suggests that many rare variants can contribute to the phenotypic variation [4,5], association studies to detect rare alleles have become more and more important. In this study, we try to answer two questions: (1) Within a region in the genome, how well do common SNPs tag the presence of rare alleles? (2) When selecting common tagging SNPs for association studies to detect rare alleles, should we exclude SNPs in strong LD with each other (*r*^2^ > 0.95), or does it help to capture more information on the rare alleles if we include tagging SNPs in strong LD (*r*^2^ > 0.95) with each other? To answer the first question, we analyzed the correlation between common SNPs and the number of rare alleles in samples of rare SNPs (i.e., SNPs containing rare alleles) in each region of the chromosomes. Then, for the second question, we studied the change in correlation between a single rare SNP and common tagging SNPs that is achieved by including SNPs in strong LD with each other when selecting common tagging SNPs.

## Methods

### Sample

We use the Genetic Analysis Workshop 17 (GAW17) data set, which is composed of 697 individuals in this study. The data include 24,487 SNPs, 74% (18,131) of which are considered rare SNPs with a minor allele frequency (MAF) less than 0.01 and only 12.8% of which are common SNPs with MAF > 0.05. Because of the unbalanced number of rare and common SNPs in the data, in order to study the capability of the common SNPs to tag rare variants, we incorporate into this data set genotype data from the International HapMap Project, release 28 (http://hapmap.ncbi.nlm.nih.gov/). The final data set includes 627 individuals from 7 populations: European (88), Chinese (91), Chinese in Denver (90), Japanese (92), Luhya (98), Tuscan (61), and Yoruba (107). After removal of SNPs in perfect LD, we are left with 13,777 rare SNPs (MAF < 0.01) and 116,944 common SNPs (MAF > 0.05).

### Correlation between common SNPs and the presence of rare alleles

We divide the genome into nonoverlapping 1-Mb bins. For each bin, we separate the rare SNPs from the common SNPs. The common SNP value for each individual is the number of minor alleles. The correlation between the set of common SNPs and the numbers of rare alleles is calculated in each bin as follows. For *n_s_* randomly selected rare SNPs (here we studied *n_s_* = 5) in a bin, we quantify the number of rare alleles as the total number of rare alleles, *y_i_*, that individual *i* (*i* = *1*, *2*, *…*,*N*) carries. The correlation between the variable *y_i_* and the common SNPs in the bin is calculated over the *N* individuals in two ways. In the first way we calculate the Pearson correlation *r* between *y_i_* and each of the common SNPs, taking the maximum *r*^2^. In the second way we calculate the multiple correlation *R*^2 ^[6] between *y_i_* and the common SNPs, using a multiple regression model. These two correlations are calculated for each consecutive region across the whole genome. We repeat the random sampling of the rare SNPs and the calculation of the correlation *n_r_*/*n_s_* times (i.e., the closest integer to *n_r_*/*n_s_* ) if *n_r_* >*n_s_*, where *n_r_* is defined as the total number of rare SNPs in a bin.

We calculate the correlations between the common SNPs and the number of rare alleles in rare SNPs separately in each of the seven subpopulations, to test whether the tagging capability is different in different populations. We also calculate the correlation between common SNPs and the number of each of two types of rare alleles (synonymous and nonsynonymous) to test whether common SNPs have a different capability to tag these two types of rare alleles.

To examine whether the correlations between common SNPs and rare alleles are due to statistical noise, we perform a permutation test. We permute each of the common SNPs within the bin across individuals and calculate the correlations between the variable y_i _and the permuted common SNPs. Then the observed and permutation correlation distributions are compared using a Kolmogorov-Smirnov test. We also compare the means of the two distributions using a *t* test.

### Capability of common SNPs in strong LD to capture rare alleles

We hypothesize that incorporating common SNPs in strong LD will capture significantly more variation resulting from rare alleles than using only the common SNPs in less strong LD with each other. We select the common SNPs within a 1-Mb region of each rare SNP and divide them into two sets. The first set is composed of the common tagging SNPs with LD of *r*^2^ ≤ 0.80 between each pair; we call this set A. The second set is composed of the common SNPs with LD of *r*^2^ ≤ 0.95 between each pair, which we call set B. So set B has two parts: all the SNPs in set A (*r*^2^ ≤ 0.80) and those SNPs in set (B − A) that are in higher LD with the SNPs in set A or between themselves (0.80 <*r*^2^ ≤ 0.95). Any SNP in perfect LD (*r*^2^ = 1) with another is excluded from the data. Then we calculate the multiple correlations *R*^2^[6] between each rare SNP and the set of common SNPs (set A and set B, respectively). Because *R*^2^ always increases when the number of independent variables in the model increases,  is always greater than or equal to [6], where the subscripts A and B represent set A and set B, respectively.

An *F* statistic,(1)

where *n_A_* and *n_B_* are the numbers of SNPs in set A and set B, respectively, is calculated to test whether the increase in  over  to predict the rare alleles is significant. Because *R*^2^ increases with the number of explanatory terms in a model, we use the adjusted , which adjusts for the number of explanatory common SNPs in the multiple regression model [6], to evaluate the multiple correlation:(2)

where *n* is *n_A_* or *n_B_*.

In order to test whether the increase in *R*^2^ is due to the stronger LD among the SNPs in set B, which comes from the SNPs in set (B − A), or due to the larger number of SNPs from set (B − A), we evaluate the significance of the *F* statistic by comparison to a sample of 1,000 replicates of its permutation distribution, obtained by permuting across individuals the set of SNPs in set B but not in set A (i.e., the SNPs in set (B − A)), which breaks any LD structure between sets A and (B − A) but keeps the structure within the set (B − A).

For each rare SNP, we also compare its multiple correlation  with the common SNP set A having LD given by *r*^2^ ≤ 0.95 and with set B having LD given by *r*^2^ ≤ 0.99.

## Results

### Correlation between the number of rare alleles and common SNPs within a region

**Figure 1:**
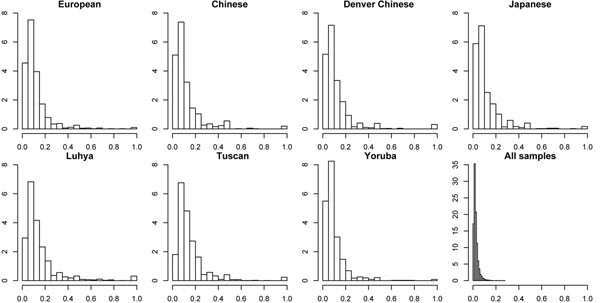
**Distribution of the correlation *r*^2^ between rare alleles and common SNPs in the subpopulations and overall.** The correlation is between the common SNPs and the number of rare alleles present in five random rare SNPs within a 1-Mb region. X-axes are the correlation r^2^, y-axes are the probability densities.

Using all 627 samples, the correlation between the number of rare alleles in any randomly selected five rare SNPs and a set of common SNPs within a 1-Mb region is less than 0.1 for both correlation measures. The correlation between the number of rare alleles and a set of common SNPs within subpopulations was larger than that of the samples overall (Table 1; Figure 1). The mean adjusted multiple correlation  for European, Chinese, Denver Chinese, Japanese, Luhya, Tuscan, and Yoruba ranged from 0.06 to 0.24 (Table 1). Compared with random correlations, which are given by correlations between the number of rare alleles and a set of randomly permuted common SNPs, there was no significant difference in the total sample. In the subpopulations, however, the correlations between the number of rare alleles and the set of common SNPs were significantly different from random correlations (*P* < 0.001) (Table 1), but the difference was quite small.

**Table 1:** Mean multiple correlation  between (1) the set of common SNPs and the number of rare alleles, (2) permuted common SNPs and the number of rare alleles, (3) the set of common SNPs and the number of synonymous rare alleles, and (4) the set of common SNPs and the number of nonsynonymous rare alleles

Population	(1) Common vs. rare SNPs	(2) Random correlation	*t* test *P*	Kolmogorov-Smirnov test *P*	(3) Common vs. synonymous rare SNPs	(4) Common vs. nonsynonymous rare SNPs	*t* test *P*
European	0.078	−0.022	2.84 × 10^−9^	9.66 × 10^−15^	0.078	0.077	0.952
Chinese	0.067	0.003	6.80 × 10^−6^	1.28 × 10^−5^	0.090	0.041	0.024
Denver Chinese	0.063	−0.002	2.30 × 10^−6^	3.052 × 10^−10^	0.085	0.064	0.350
Japanese	0.089	0.004	1.50 × 10^−8^	6.17 × 10^−12^	0.091	0.081	0.668
Luhya	0.238	−0.0006	<2.2 × 10^−16^	<2.2 × 10^−16^	0.241	0.233	0.678
Tuscan	0.063	−0.002	0.001	4.60 × 10^−6^	0.088	0.045	0.100
Yoruba	0.120	−0.007	<2.2 × 10^−16^	<2.2 × 10^−16^	0.142	0.099	0.008
All samples	0.053	0.054	0.580	0.118	0.057	0.048	7.74 × 10^-4^

In the total sample, the set of common SNPs has a correlation with the number of rare synonymous alleles  and with the number of rare nonsynonymous alleles ; the difference, although small, is significant (*P* = 7.74 × 10^−4^). In the subpopulations, the set of common SNPs also showed higher correlations with the number of rare synonymous alleles than with the number of rare nonsynonymous alleles, and the difference was most significant in Yoruba (*P* = 0.008). Note that in Yoruba, although the average correlation between common SNPs and the number of rare alleles is not high , it is significantly different from a random correlation, which suggests that common SNPs are able to capture some information on the number of rare alleles. In Yoruba, the set of common SNPs has a significantly smaller correlation with the number of rare synonymous alleles than with the number of rare nonsynonymous alleles (*P* = 0.008), which may indicate that the common SNPs are more prone to detecting nonfunctional SNPs than functional SNPs in this population. The correlation between common SNPs and the number of rare alleles is highest in Luhya , but common SNPs show no significant difference in capturing synonymous and nonsynonymous SNPs.

### Capability of common SNPs in strong LD to capture rare variants within a region

**Figure 2:**
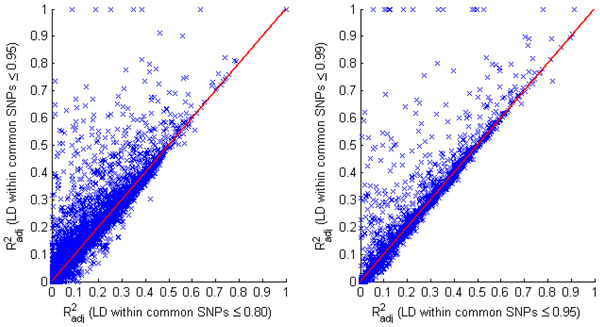
**Distribution of the multiple correlation *R*^2^ between a rare SNP and a set of common SNPs within a 1-Mb region of the rare SNP.** Each point represents a rare SNP. The *x*-axis is the adjusted  between the rare SNP and the common SNPs in set A, and the *y*-axis is the adjusted  between the rare SNP and the common SNPs in set B. SNPs in set B have stronger LD than SNPs in set A, thus set B contains all the SNPs in set A and the SNPs that have stronger LD with those in set A or between themselves. In the left-hand panel, SNPs in set A have LD *r*^2^ ≤ 0.8 and SNPs in set B have LD *r*^2^ ≤ 0.95. In the right-hand panel, SNPs in set A have LD *r*^2^ ≤ 0.95 and SNPs in set B have LD *r*^2^ ≤ 0.99.

By comparing two correlations—the adjusted multiple correlation between a rare SNP and the set of common SNPs in set A (LD of *r*^2^ ≤ 0.80) and the adjusted multiple correlation between that rare SNP and the set of common SNPs in set B (composed of both the SNPs in set A with LD *r*^2^ ≤ 0.80 and the SNPs in stronger LD, 0.80 <*r*^2^ ≤ 0.95)—we found that some rare SNPs showed higher correlations with the common SNPs in set B than with those in set A (Figure 2). The distributions of the two correlations are significantly different using a Kolmogorov-Smirnov test (*P* = 2.44 × 10^−6^), although their means are not significantly different by a *t* test (*P* = 0.07). If set A is the set of common SNPs with LD ≤ 0.95 and set B is the set of common SNPs with LD ≤ 0.99, then set B also shows higher correlation with some rare SNPs than set A does, and the difference of the distributions of the two  values is significant (Kolmogorov-Smirnov test *P* = 0.02). We used the *F* statistic to evaluate whether the increase in *R*^2^ for set B is due to the extra SNPs in stronger LD in set B or is just due to chance. For the points in Figure 2 that show an increase in  greater than 0.30, most of the increases are significant (nominal *P* < 10^−5^ using an *F* test that assumes normality; *P* < 0.03 by permutation), except for two points (nominal *P* > 0.08, permutation *P* > 0.11).

## Discussion

In this study, we found that within a region in the genome, overall the common SNPs are not highly correlated with the number of rare alleles, so they are not powerful for tagging the presence of rare alleles. But in subpopulations, the common SNPs can capture some information on the presence of rare variants, and their increased correlations are statistically significant but are often small (Table 1). We also found that including tagging SNPs in strong LD with each other is helpful in detecting rare alleles.

Common SNPs have higher correlations with the presence of rare SNPs in the subpopulations, which indicates that population structure influences the tagging power. The common SNPs have lower correlations with the presence of nonsynonymous SNPs, especially in the Yoruba population, which may indicate difficulty in capturing rare functional variants in that population. In addition to the presence of rare alleles, we also analyzed the correlation between common SNPs and another variable, a collapsing statistic for rare SNPs [7-9], which has the value 1 if a rare allele is present and the value 0 if no rare alleles are present among several randomly selected SNPs within a genome region. We obtained similar results with the collapsing variable (data not shown).

Our study suggests that we should not exclude SNPs in strong LD (e.g., *r*^2^ > 0.95) from tagging SNPs in an association study, because they can help to detect rare SNPs. They are less helpful for predicting disease risk, however, because their attributable risk is so small; but the significant associations detected by them could be important for detecting new metabolic pathways.

The multiple correlation *R*^2^ could be overadjusted because the adjusting assumes independence of the common SNPs, which is not the case for our study. But we nevertheless get increased  to tag rare SNPs by including SNPs in strong LD with each other among the tagging SNPs, which indicates their importance in an association study to detect causal variants.

## Conclusions

In this study, we found that, overall, common SNPs are not good at capturing the presence of rare alleles within a region of the genome, but they can capture some information on their presence in subpopulations. The common SNPs are more prone to capturing nonfunctional rare SNPs, especially in some populations. We also found that including tagging SNPs in strong LD with each other can be helpful in detecting rare variants.

## Competing interests

The authors declare that they have no competing interests.

## Authors’ contributions

XZ and RCE conceived of the study and participated in its design and coordination, XS performed the statistical analysis and drafted the manuscript. JN prepared data from the HapMap Project. RCE, XZ and JN helped to draft and modify the manuscript. All authors read and approved the final manuscript.
